# Dual Action of BPC194: A Membrane Active Peptide Killing Bacterial Cells

**DOI:** 10.1371/journal.pone.0061541

**Published:** 2013-04-19

**Authors:** Gemma Moiset, Anna D. Cirac, Marc C. A. Stuart, Siewert-Jan Marrink, Durba Sengupta, Bert Poolman

**Affiliations:** 1 Departments of Biochemistry and Biophysical Chemistry, Groningen Biomolecular Sciences and Biotechnology Institute (GBB) and Zernike Institute for Advanced Materials, University of Groningen, Groningen, The Netherlands; 2 Institute of Computational Chemistry, University of Girona, Campus Montivili, Girona, Spain; 3 Physical Chemistry Division, CSIR-National Chemical Laboratory, Pune, India; Nagoya University, Japan

## Abstract

Membrane active peptides can perturb the lipid bilayer in several ways, such as poration and fusion of the target cell membrane, and thereby efficiently kill bacterial cells. We probe here the mechanistic basis of membrane poration and fusion caused by membrane-active, antimicrobial peptides. We show that the cyclic antimicrobial peptide, BPC194, inhibits growth of Gram-negative bacteria and ruptures the outer and inner membrane at the onset of killing, suggesting that not just poration is taking place at the cell envelope. To simplify the system and to better understand the mechanism of action, we performed Förster resonance energy transfer and cryogenic transmission electron microscopy studies in model membranes and show that the BPC194 causes fusion of vesicles. The fusogenic action is accompanied by leakage as probed by dual-color fluorescence burst analysis at a single liposome level. Atomistic molecular dynamics simulations reveal how the peptides are able to simultaneously perturb the membrane towards porated and fused states. We show that the cyclic antimicrobial peptides trigger both fusion and pore formation and that such large membrane perturbations have a similar mechanistic basis.

## Introduction

Membrane active peptides (MAPs) represent a class of molecules that are able to interact with membranes, leading to fusion, poration and/or translocation. Depending on their mode of action, these peptides have been traditionally classified in three different categories: fusogenic peptides, antimicrobial peptides and cell-penetrating peptides [1]–[4]. More and more data suggest that this classification is too rigid as some peptides have multiple functionalities [5]–[14]. For example, both fusogenic and antimicrobial peptides have been shown to induce leaky fusion in vesicles [6], [15], [16], and a cell-penetrating peptide has been shown to induce leaky fusion of liposomes [7]. For antimicrobial peptides it has been speculated that this “multihit mechanism” increases their potency [17], [18]. Despite much progress in the characterization of peptide-membrane interactions, the molecular details of the events leading to membrane fusion, poration, and peptide translocation are still poorly understood. A powerful tool to study peptide-membrane interactions at the molecular level is the molecular dynamics (MD) technique [19]–[22].

Here, we combine MD simulations with a number of experimental techniques, including Dual-Color Fluorescence Burst Analysis (DCFBA), Förster Resonance Energy Transfer (FRET) and cryogenic Transmission Electron Microscopy (cryo-TEM), to explore the process by which peptides are able to act on a membrane in a dual way. Moreover, we relate our findings to cryo-TEM studies in Escherichia coli cells. The peptide investigated, BPC194: c(KKLKKFKKLQ), is a cyclic antimicrobial peptide that adopts a β-sheet structure upon interaction with the membrane [23]. The peptide was selected from a library of de novo synthesized head-to-tail cyclic peptides [24], [25], which showed a high antimicrobial activity towards Gram-negative plant pathogenic bacteria like Erwinia amylovora, Pseudomonas syringae and Xanthomonas vesicatoria. We have previously probed the pore forming propensity of this peptide and showed that the β-conformation of the peptide is optimal for the stabilization of the curvature of the transmembrane pore [26].

We show here how BPC194 also induces membrane fusion, probing the process at an atomistic, molecular and ensemble level. Two seemingly unrelated processes: pore formation and membrane fusion are shown to occur simultaneously and influence the paths of both modes of action. Our in silico and in vitro observations correlate with in vivo data and provide a mechanistic framework for growth inhibition of bacterial cells by BPC194.

## Materials and Methods

### Reagents and Apparatus

The 2-(4-(2-hydroxyethyl)-1-piperazinyl)-ethanesulfonic (HEPES) was from Roche Diagnostics GmbH; 1,2-dioleoyl-sn-glycero-3-phosphatidylglycerol (DOPG) was from Avanti Polar Lipids; 1,1′-dioctadecyl-3,3,3′,3′-tetramethylindodicarbo-cyanine perchlorate (DiD), 3 kDa dextran-fluorescein, N-(7-nitrobenz-2-oxa-1,3-diazol-4-yl)-1,2-dihexadecanoyl-sn-glycero-3-phosphoethanolamine (NBD-PE) and Lissamine™ Rhodamine B 1,2-dihexadecanoyl-sn-glycero-3-phosphoethanolamine (Rh-DHPE) were from Invitrogen. For in vivo experiments, the medium used was Luria Broth (10 g/L Bacto Tryptone (Becton Dickinson), 5 g/L Yeast extract (Becton Dickinson) plus 10 g/L NaCl; Merck). The buffer used for cell imaging with the light microscope was 10 mM sodium phosphate, pH 7.5, containing 150 mM NaCl. For cryo-TEM assay with E. coli cells we used the buffer 120 mM potassium phosphate, pH 7.0, which has an osmolality equal to that of LB (measured by determination of the freezing point in an Osmomat 030, Gonotec) or sodium phosphate buffer (same for light microscopy). In vitro solutions were prepared in 10 mM HEPES-NaOH, pH 7.2, containing 150 mM NaCl (the so-called physiologic ionic strength). The peptides BPC194, c(KKLKKFKKLQ), and its linear counterpart BPC193, H-KKLKKFKKLQ-OH, were synthesized as described previously (23) and purified by reverse-phase preparative HPLC (purities >95%).

### Strains, Growth and Cell Imaging

Escherichia coli (E. coli) K-12 strain MC1061 [27] was grown from single colonies in LB at 37°C under vigorous aeration until the culture reached an OD_600_ of 0.15 for light microscopy and 0.6 for electron microscopy. Prior to the light microscopy, 1 mL of cells was washed twice with fresh LB medium and the pellet was finally resuspended in a 400 µL of LB to get an optimal cell density. Afterwards, 1.5% agarose solution in LB was pipetted onto a multispot microscope slide of 12 wells (Hendley-Essex). The coated slide was left to solidify at 4°C for 15 min. A 1 µL drop of cell suspension mixed 1∶1 (v/v) with buffer or peptide solution (final concentration range from 0.75 µM to 100 µM) was placed on each well and immediately covered with a coverslip. Cells were imaged for approximately 4 hours using Differential Interference Contrast (DIC) transmitted-light in an inverted microscope Observer Z1 (Carl Zeiss), equipped with a Zeiss LCI Plan-NeoFluar 63× objective (numerical aperture of 1.3) and a Cool-Snap HQ2 CCD camera (Photometrics). Cell growth rates were calculated from the increase in cell number over time; growth rates in liquid LB medium and LB-agarose were comparable. For electron microscopy, the cells were centrifuged (4 min; 10,000×g; room temperature) and concentrated to a final OD_600_ of 100.

### Preparation of Lipid Vesicles

Large unilamellar vesicles (LUVs) were prepared as described elsewhere [26]. Briefly, rehydration of a dried DOPG lipid film was done in 10 mM HEPES-NaOH, 150 mM NaCl, pH 7.2. Vesicles were then subjected to five cycles of flash freezing in liquid nitrogen and rapid thawing at 37°C. Subsequently, liposomes were extruded 11 times through a 200 nm polycarbonate filter (Avestin).

For the DCFBA experiments the lipid film was made by mixing the membrane dye DiD with DOPG lipids at a molar ratio of 1∶12,000 (corresponding to ∼15 molecules of DiD per liposome for vesicles with a diameter of 200 nm) and the rehydration was done in the presence of the lumen cargo molecule: 3 kDa dextran labeled with fluorescein (5 µM). Liposomes were separated from the non-encapsulated fluorophores by centrifugation (20 min; 270,000×g; 20°C) and resuspended in the buffer to a final lipid concentration of 80 µM.

For the FRET assays, the lipid film contained 1 mol% NBD-PE and/or 1 mol% Rh-DHPE, and the final lipid concentration was 125 and 250 µM DOPG.

For cryo-TEM, the liposomes were briefly sonicated before extrusion to increase the unilamellarity of the vesicles (5 pulses of 1 sec. at 75% amplitude with a Sonics Vibra Cell VCX 130 sonicator) and the final lipid concentration was 5 mM DOPG.

### DCFBA

In the DCFBA experiment, liposomes were labeled with two, spectrally non-overlapping fluorescent probes [28]. The DiD probe was incorporated in the phospholipid bilayer, while the fluorescein-labeled dextran filled the aqueous interior of the liposomes. By using a dual-color laser-scanning microscope, we monitored membrane-disrupting effects at the single liposome level. Different amounts of peptide (0 to 27 µM) were added to 80 µM DOPG liposomal solutions, yielding total peptide-to-lipid (P/L) ratio from 0 to 0.3. The samples were equilibrated for 10 minutes at room temperature after each addition of peptide. The fluorescence bursts, resulting from the diffusion of the liposomes through the detection volume, were measured for 10 min. The internal cargo concentration (C) of the i^th^ liposome (burst) is given by:
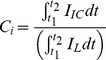
where I_L_ is the fluorescence intensity of the lipid marker, DiD, above a certain threshold between t_1_ and t_2_. I_IC_ is the fluorescence of the internal cargo. The average concentration of internal cargo, C_av_, over all the liposomes can be obtained from C_i_:



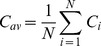
where N corresponds to the number of liposomes [28]. Samples were imaged on a commercial laser-scanning confocal microscope, LSM 710 (Carl Zeiss MicroImaging, Jena, Germany), using an objective C-Apochromat 40×/1.2 NA, a blue argon ion laser (488 nm) and a red He-Ne laser (633 nm).

### FRET

Fusion was monitored using the Förster resonance energy transfer (FRET)-based methodology described before by others [29], [30], using a Cary Eclipse fluorescence spectrophotometer (Varian). Two different FRET assays were performed, i.e., positive- and negative-FRET. In positive-FRET, DOPG vesicles labeled with 1 mol% NBD-PE (donor) were mixed with DOPG vesicles labeled with 1 mol% Rh-DHPE (acceptor) at a molar ratio of 1∶1. If fusion occurs, the lipids of the donor and acceptor vesicles will mix and the FRET efficiency, as monitored by an increase in the acceptor emission (intensity at λ_em_ = 590 nm, Rhodamine emission), will increase. On the other hand, in the negative-FRET assay, DOPG vesicles labeled with both 1 mol% NBD-PE and 1 mol% Rh-DHPE were mixed with unlabeled vesicles at a molar ratio of 1∶3. In this case, as the vesicles fuse, the average distance between donor and acceptor increases. Thus, the FRET efficiency decreases proportionally and is monitored by an increase in the donor emission (intensity at λ_em_ = 530 nm, NBD emission). In both assays the peptide BPC194 was added to a final concentration in the range of 0 to 200 µM. The absorbance peaks of samples were kept <0.1, and various controls were done to minimize the inner filter effects (Fig. S1A–B in Supporting Information S1). Quantification of fusion was calculated from the increase in NBD emission in the negative-FRET assay at two different lipid concentrations (125 and 250 µM). The 0% fusion was taken from the intensity of free-peptide vesicles. The other end of the fusion scale (100%) was calculated by adding CaCl_2_ (194 mM) as a fusogenic agent to the vesicle suspension [29]. Control experiments to correct the intensities for quenching of NBD and Rhodamine upon peptide and calcium addition were also performed (Fig. S1C–F in Supporting Information S1).

### Cryo-TEM

Samples for cryo-TEM were prepared by deposition of a few µL of vesicle solution (with buffer or peptide at a final P/L ratio of 0.01) or cell solutions (mixed 1∶1 (v/v) with either buffer for the control or BPC194 to a final P/L ratio of 0.02 or 0.4) on holey carbon-coated grids (Quantifoil 3.5/1, Quantifoil Micro Tools). After blotting the excess liquid, the grids were vitrified in liquid ethane in a Vitrobot (FEI) and transferred to a Philips CM 120 cryo-electron microscope equipped with a Gatan model 626 cryo-stage, operating at 120 kV. Images were recorded under low-dose conditions with a slow-scan CCD camera. For the in vivo system, images were taken at two time points, one or 30 minutes after mixing with peptide. For the in vitro system, images were taken 10 minutes after mixing with peptide and a total of 98 cells were analyzed in the presence of peptide and 58 cells without peptide.

### Simulations System Set-up

The starting system consisted of two solvated DPPG (dipalmitoyl-phosphatidylglycerol; anionic lipid) bilayers in the fluid phase, comprising of 512 lipids each and placed at a distance of about 3 nm from each other. The DPPG lipids and their palmitoyl tails are well characterized in our group and have been used in our previous study on pore formation by BPC194 [23], [26]. We emphasize that this lipid is in the fluid phase at conditions in the molecular dynamics simulations. BPC194 peptides were placed between the bilayers in the water phase at a P/L ratio of 1∶15. The cyclic peptides were modeled based on a previous study [23], [26]. The system consisted of about 32000 water molecules. K^+^ ions were added as counter-ions for anionic lipids and Cl^−^ ions were added to neutralize the overall system. Five simulations were set up with different starting random velocities to obtain statistically significant results. A pure DPPG bilayer was also simulated for reference. Furthermore, a simulation where the inactive linear analog (BPC193) was tested at the same conditions as the active cyclic peptide was performed as a control. The linear peptide was modeled based on our previous study [23], [26] with charged termini to best represent the experimental conditions. For an overview of the simulations see Table 1.

**Table 1:** Overview and statistics of the MD simulations.

Simulation	Time (ns)	Nr. Pores	Time scale Pore (ns)	Tilt >85°	Splay >170°
Pure DPPG	100	–	–	0	2
F1	360	2	40/58	24	9
F2	210	–	–	18	8
F3	590	1	150	28	12
F4	160	2	10/70	10	6
F5	230	–	–	16	10
Average	–	–	–	19.2±3.1	8.9±1.1

The percentage of lipids in the contacting monolayers which, during the simulation, tilt by more than >85° or splay by more than >170° is indicated. The standard error of the average is obtained from the standard deviation between all five simulations. The simulation length and formation of pores is also indicated.

### Simulations Parameters

The GROMACS software package [31] was used to perform all MD simulations. The GROMOS force-field 43a2 [32] was used to describe the peptide and peptide-solvent interactions. The force-field for DPPG lipids was taken from a previous study [23], [26]. All force-fields were parameterized for use with a group-based twin range cut-off scheme (using cutoffs of 1.0/1.4 nm and a pair-list update frequency of once per 10 steps), including a reaction field (RF [33]) correction with a dielectric constant of 78 to account for the truncation of long-range electrostatic interactions. The water was modeled using the SPC model [34]. A time step of 2 fs was used. Bond lengths were constrained using the LINCS algorithm [35]. The simulations were performed in the NP_|_P_Z_T ensemble using periodic boundary conditions. The temperature was weakly coupled (coupling time 0.1 ps) to T = 320 K using the Berendsen thermostat [36]. The pressure was also weakly coupled (coupling time of 1.0 ps and compressibility of 4.5×10^−5^), using a semi isotropic coupling scheme in which the lateral (P_|_) and perpendicular (P_Z_) pressures were coupled independently at 1 bar, corresponding to a tension-free state of the membrane. The simulation setup is similar to that used in previous studies of peptide-membrane interactions [23], [26], [37]–[39].

### Characterization of Lipid Tilting and Splaying

The tilt of the lipids was calculated by the angle between the vector of three atoms (P, C2A and C2P) and the *z*-axis as a reference. The values close to 0° mean no tilt whereas values close to 90° mean complete tilt. The splay of the lipids was calculated by the angle between the vectors of two atoms of one lipid tail (C2A, C2P) and the other lipid tail (C1A, C1P). The values close to 180° indicate the splay of the hydrocarbon tails.

## Results

### Cell Growth Inhibition and Cell Envelope Defects by BPC194

**Figure 1:**
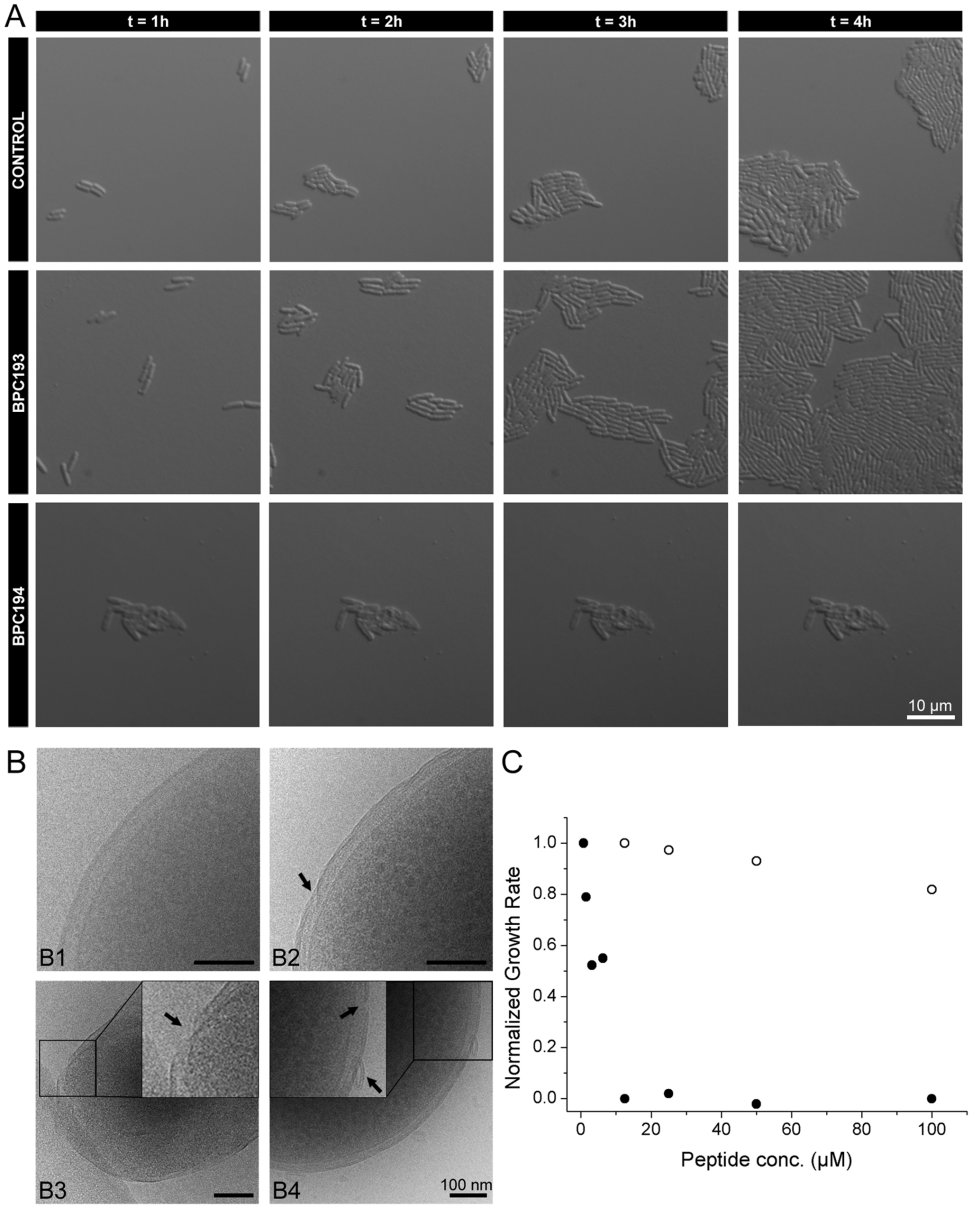
Growth inhibition and cell envelope defects by BPC194. A. Cell growth imaging of E. coli without peptide (control) and with 12.5 µM of BPC193 or BPC194 during four hours. Note that the first image was taken after 1 h. and one or two cell divisions had already taken place in the control and BPC193 samples. B. Cryo-TEM micrographs of E. coli cells without (B1) and with BPC194 (B2–B4). Black arrows point out severe disruption of the cell envelope: membrane irregularities (B2); putative contact sites between IM and OM (B3); rupture of the cell envelope and budding off of vesicle-like structures (B4). Scale bars represent 100 nm. C. Cell growth rates of E. coli in the presence of different concentrations of BPC194 (full circles) and its linear analog, BPC193 (empty circles). The values are normalized to the growth rate in the absence of peptide, which corresponded to 1.15 h^−1^.

To analyze the mechanism of action of BPC194, we monitored the aggregation, growth and morphology of E. coli cells by light microscopy and used the linear analog of BPC194, which lacks activity, as a control. Figure 1A summarizes the results of the cell growth over 4 hours of imaging (see also Movies S1, S2, S3). The linear analog, BPC193, did not effect the cell growth up to a concentration of at least 100 µM. BPC194 already inhibited the growth at an order of magnitude lower concentration. The growth rate of E. coli as a function of peptide concentration is shown in Fig. 1C. Unlike the linear peptide, the cyclic peptide caused severe inhibition of growth and aggregation of the cells. This is a remarkable difference because both peptides have identical sequence and overall charge (+6 at physiological pH). To determine whether or not the different effects of BPC194 and BPC193 are caused by (partial) degradation of the peptides by *E. coli* cells, we tested their stability (see Supporting Methods in Supporting Information S1). The fate and concentration of the peptides was followed by reverse-HPLC. The results show that there is no observable degradation of the peptides after 1 h of incubation with cells (or even cell lysates), that is, under conditions that BPC194 is completely inhibiting growth and BPC193 is having no effect (Fig. S2 in Supporting Information S1). To investigate further the effect of the cyclic peptide on the ultrastructure of the cells, we performed cryo-TEM (Fig. 1B). BPC194 caused disruption of the cell envelope (shown in black arrows) in all the cells analyzed, most notably the integrity of the inner and outer membrane (IM and OM) was disrupted. The cell envelope is no longer smooth (panel B2) with the presence of contact sites between the IM and OM (panel B3) in about 30% of the cells, and in some regions the membrane was pinched off or budding off of vesicle-like structures was observed (panel B4).

The results show that only the cyclic peptide is able to abolish cell division, which is preceded by cell aggregation. The positive charge of the peptide is not sufficient for cell aggregation and subsequent disruption of the cell envelope, as the linear analog does not show similar effects. The locked cyclic conformation of BPC194 might be the key factor in the initial interaction with the cell envelope, causing large physical stress and damage, what leads to inhibition of cell division and ultimately to cell death.

### Simultaneous Pore Formation and Fusion Action of BPC194

**Figure 2:**
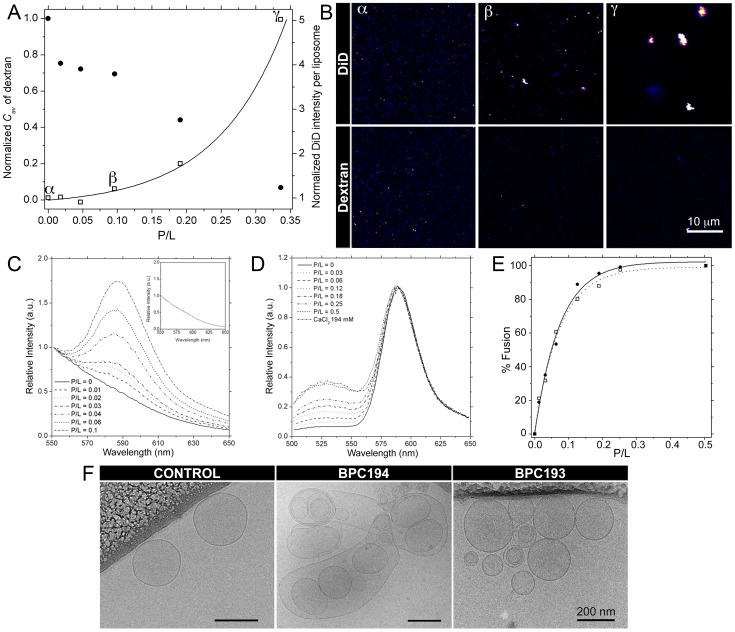
Simultaneous pore formation and fusion activity of BPC194. A: The normalized concentration of dextran inside the liposomes, C_av_, (filled circles) and the normalized intensity of membrane-associated DiD per liposome (empty squares) at different P/L ratios. B: Confocal images of the lipid vesicles in the DiD and dextran detection channel at three different P/L ratios; α, P/L = 0; β, P/L = 0.1; and γ, P/L = 0.3. C: Positive-FRET upon peptide addition. The emission of Rhodamine increases due to vesicle fusion. Inset: Controls done with the ‘inactive’ linear analog of BPC194, that is, BPC193 at the same peptide concentrations. D: Negative-FRET upon peptide addition. The emission of NBD increases due to a decrease in FRET efficiency as a result of vesicle fusion. E. Quantification of fusion at different P/L ratios and at two different lipid compositions, 125 µM (full circles) and 250 µM (empty squares). D. Representative cryo-TEM micrographs of DOPG vesicles without peptide (control) and with BPC194 or the linear analog BPC193.

The use of model systems is required to get fundamental chemical understanding of the mode of action of membrane-active compounds as whole cells are simply too complicated for such an analysis. We used DOPG vesicles albeit that, similar observations have been made in membranes composed of mixtures of zwitterionic and anionic lipids [23]. However, the higher the fraction of anionic lipids, the stronger the binding and poration of BPC194. We used solely negatively charged lipids as in this system the pores are stable for long periods of time (on the time scale of minutes) as inferred from electrophysiology studies [26]. DCFBA, FRET and cryo-TEM techniques were used to probe the peptide-membrane interactions of BPC194, using BPC193 as negative control. First, DCFBA experiments were performed upon addition of different amounts of peptide to the DiD-labeled vesicles, filled with the internal cargo 3 kDa dextran-fluorescein [28]. The average concentration of internal cargo and the normalized intensity of membrane-associated DiD per liposome as a function of peptide-to-lipid ratio are summarized in Fig. 2A. As the P/L ratio increases the amount of dextran inside the vesicles, C_av_, decreases, which is indicative of the poration activity of the peptide (full circles). In parallel with the cargo leakage, we observed that the amount of DiD per vesicle increased, which points towards vesicle fusion or aggregation (empty squares). The DCFBA data (Fig. 2A) in conjunction with the confocal images (Fig. 2B) confirm the two concurrent events, poration and fusion/aggregation. In panel γ, mesoscopic aggregates can be observed in the DiD channel, whereas the corresponding signal of internal cargo has disappeared due to leakage.

To distinguish between fusogenic action (lipid mixing of vesicles) and aggregation, we performed both positive-FRET (Fig. 2C) and negative-FRET (Fig. 2D) [29]. The fusogenic action of the cyclic peptide BPC194 was confirmed by both assays, thus classifying the observations of Fig. 2B γ as fusion of vesicles. Using the emission intensity of the FRET donor, NBD-PE, after correcting for peptide quenching, we quantified the percentage of fusion at each P/L ratio with two different lipid concentrations (Fig. 2E). The percentage of fusion increased with the peptide addition until a P/L of roughly 0.3, at which the fusion of vesicles as probed by FRET was maximal. At this particular P/L ratio, the cargo of the vesicles had already completely leaked out (Fig. 2A and 2B). As a control, we analyzed the inactive linear analog of BPC194, that is BPC193, at the same peptide concentrations and there was no change in the FRET efficiency (Fig. 2C; inset).

Cryo-TEM also revealed the fusogenic behavior of BPC194 and the most representative images of vesicles without peptide, with BPC194 and BPC193 are shown in Fig. 2F. Vesicles without peptide were on average about 200 nm. BPC194 yielded larger vesicles, consistent with membrane fusion, whereas the linear BPC193 brought the vesicles close together but vesicle fusion was not observed (Fig. 2F). The linear BPC193 peptide was previously shown to be inactive and vesicles aggregates were already seen by confocal microscopy [23], [26].

We note that complete fusion occurs at a bound peptide-to-lipid ratio of around 0.15. It has been shown by Melo and coworkers that the bound peptide concentrations at the MIC value are comparable to those of the thresholds effects (pore formation) in model membranes [40]–[42]. The apparent difference between the in vivo and in vitro numbers arises from the fact that in a standard MIC assay, the number of cells is very low and the lipid concentrations are in the nanomolar range. When all these factors are taken into account, the corresponding P/L ratio in the bacterial cell is around 0.1 and thus very similar to what we find in the membrane model system. In accordance, substantial fusion and leakage were observed at similar P/L ratios in the in vivo and in vitro assays. The overall data indicate that the fusogenic and poration activity of the cyclic peptide occur simultaneously and points to a “multi-hit” mechanism of action.

### Molecular Basis for Concurrent Fusion and Leakage by MD Simulations

**Figure 3:**
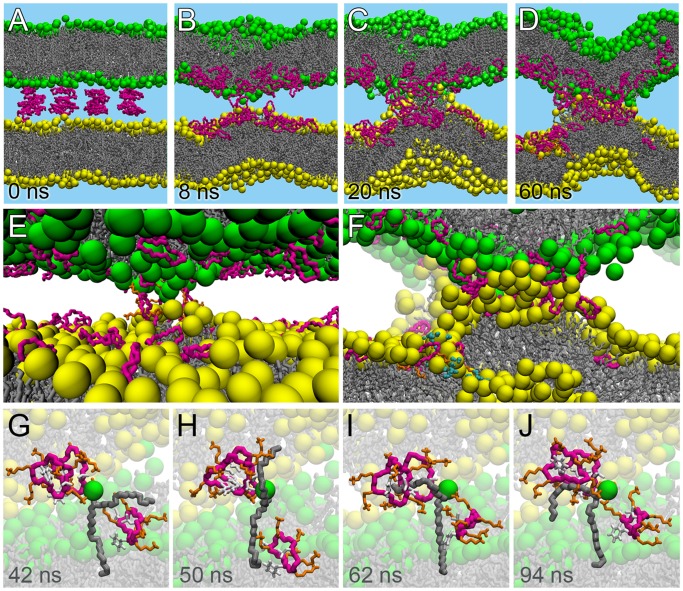
Molecular view of the sequence of events of the leaky fusogenic action of cyclic peptides. A. Initial simulation setup with peptides placed between two bilayers. B. Bridging of proximal leaflets of the two bilayers by BPC194. C. Lipid bulging caused by the action of peptides associated with the bilayers. D. Pre-stalk intermediate accompanied by disordered toroidal pore. E. Close-up of the bridging peptides. F. Close-up of the stalk-pore complex. G–J. Splaying of a lipid during the course of a simulation. The peptides are depicted in pink, the phosphorous atoms in yellow and green respectively and the lipid chains in grey. The water is not shown for clarity. In panel F, the water molecules within the pore in one of the bilayers are shown in blue. The other pore cannot be seen in the zoom-in but is visible in panel D.

To study the fusion and leakage events at an atomistic level, MD simulations of two DPPG bilayers were set up with multiple copies of the peptide placed between them (Fig. 3A). We note that in the simulations DPPG is in the fluid, liquid-disordered state. The P/L ratio was set to 1/15, i.e. at intermediate values for fusion and poration as observed experimentally. A control simulation was performed with the linear analog, BPC193, at the same P/L ratio. The linear counterpart of BPC194 is not able to form pores, as shown previously [26] or fuse the two apposed bilayers (Fig. S3 in Supporting Information S1). Five independent simulations were performed with the cyclic peptide, exploring a total time scale of more than 1 microsecond (Table 1). Representative snapshots showing the sequence of events in a particular simulation (F1, cf. Table 1) is depicted in Fig. 3B–D and the whole trajectory is shown in Movie S4. The other four simulations showed qualitatively similar behavior. The following steps were observed: i) peptide binding leading to membrane contact, ii) lipid perturbations leading to membrane bridge formation, and iii) peptide penetration resulting in pore formation.

#### Initial membrane contact

An initial fast binding of the peptides was observed with most peptides binding to one of the proximal monolayers, typically within 10 ns, (Fig. 3B). The binding is facilitated by multiple electrostatic interactions of the positively charged lysine residues with the negatively charged head-group moieties of the PG lipids. Importantly, a few of the peptides were able to interact simultaneously with both membranes. As a result, these peptides formed a bridge between the apposing membranes. A close-up of a few of these bridging peptides is presented in Fig. 3E.

#### Membrane bridge formation

Due to the effect of the bridging peptides, some of the lipids were perturbed locally and protruded out of the membrane and interacted with the apposing membrane, creating a membrane bridge (Fig. 3C). We refer to this state as a membrane bridge since full contact between the hydrophobic tails of lipids from the contacting leaflets did not occur, which is characteristic of membrane stalks. The membrane bridge remained stable during the length of the simulation. Formation of the membrane bridge takes place on a time scale of 10–20 ns, i.e., the time required for the bridging peptides to extract the lipid tails from the contacting monolayers. The membrane bridge showed high saddle spray curvature and can be considered an intermediate towards complete stalk formation during the process of fusion.

#### Pore formation

Subsequent to the formation of the membrane bridge, spontaneous pore formation was observed (Fig. 3D). In MD simulations, pores are distinguished by a disruption of the lamellar phase and water molecules are seen to traverse the bilayer freely via the pore. Pore formation was triggered by a few of the peptides, not involved in stabilizing the membrane bridge, that insert deeper into the bilayer. We would like to point out that the pores formed in the simulations are not fusion pores, rather they are formed adjacent to the membrane bridge and mimic the stalk/pore complex described in Ref. 50 and 60. Whether or not pores are actually formed appeared to be a stochastic process, with three out of five simulations showing pore formation (Table 1). In two of the simulations even two pores were formed. In each case, the pores formed adjacent to the membrane bridge, on a time scale ranging between 10 to 150 ns. A close-up of the membrane bridge and pore complex is shown in Fig. 3F. The pores formed are reminiscent of the disordered toroidal type as shown in previous simulation studies for this peptide [26] and other antimicrobial peptides [37], [38].

To further characterize the perturbing effect of the peptides on the lipids, we quantified the splaying and tilting of the lipid tails (see methodology for details). The results are given in Table 1. In each of the five simulations, the tilting is quite substantial compared to a pure bilayer. For example, in simulation-F3, 28% of the lipids of the proximal leaflet tilted more than 85° from their initial position at least once during the simulation. In a reference simulation of a pure DPPG bilayer, no such extensive tilting is observed. In addition, several lipids were also significantly splayed to values larger than 170°, implying a full opening of the lipid tails. Not surprisingly, the lipids that are most perturbed are the lipids in direct contact with the peptides, and especially the lipids involved in the formation of the membrane bridge, and later the pore. An example is presented in Fig. 3G–J, showing how a single lipid is splayed by interacting with a few neighboring peptides (see Movie S5). In this particular example, the splayed lipid tail remains stable over 50 ns. The stabilizing contacts between the peptides and the lipids are formed between the lysine residues and the charged head-groups, and the apolar phenylalanine and leucine residues shielding the hydrophobic tails of the lipids from the water.

## Discussion

On the basis of the in vivo and in vitro experimental data as well as the molecular dynamics simulations, we show how a synthetic cyclic antimicrobial peptide, BPC194, operates by dual action. Two seemingly different mechanisms are shown to occur concurrently, namely membrane poration and fusion. The mechanism is similar to the leaky fusion mechanism but follows a different pathway since complete leakage of vesicular content is seen and pore formation is independent of fusion. Despite the differences and higher complexity of the whole cell system as compared to the membrane model system, aggregation phenomena were observed concomitant with the inhibition of growth while membrane rupture was demonstrated by cryo-TEM. Although we did not observe genuine fusion intermediates between the IM and OM in our cryo-TEM studies of E. coli cells, we note that in Gram-negative bacteria those membranes are in close contact (5–20 nm apart) [43] and both were obstructed or brought close to each other at the point where BPC194 inhibited growth. BPC194 will not fuse bacteria together but the ability to fuse membranes may increase the potency of the peptide in cell membrane permeation of Gram-negative bacteria, i.e. when the IM and OM are in close contact.

Based on our atomistic molecular dynamics simulations and biophysical characterization, we propose a mechanism by which the peptides perform their dual action. Firstly, the peptides bind to the membrane/water interface. Both electrostatic interactions between the lysine residues and the lipid head groups and the partitioning of the hydrophobic side chains into the lipid bilayer stabilize this binding mode. In our previous work, we showed that the cyclic peptide, binds stronger compared to its linear analog, due to its pre-folded amphipathic conformation [23]. When two membranes are present in close proximity as in our current simulation setup, the peptides are actually able to bridge the two membranes. Being able to keep two liposomes at close distance is likely a necessary condition for fusion, but not sufficient, as the linear analog can induce aggregation but not fusion (Fig. 2C, Fig. 2F and Fig. S3 A–D in Supporting Information S1). By adsorbing at the interface the peptides exert considerable stress on the outer, contacting monolayers, which can be rationalized in terms of their wedge-like shape [44]. The stress induced causes a strong disordering of the lipids in the vicinity of the peptides, leading to lipid splaying, tilting, and protrusions as evidenced by our MD simulations. Eventually, a membrane bridge is formed, in which multiple (bridging) peptides and lipids form a large protrusion connecting the two apposing monolayers. The membrane bridge (also referred to as the pre-stalk in literature [45], [46]) has been suggested to be an important intermediate in the stalk-mediated pathway to fusion. In particular, recent MD studies show that at least under conditions of low hydration, early membrane fusion kinetics is not determined by the stalk energy but by the energy of pre-stalk transition states involving solvent-exposed lipid tails [45], [46]. Likewise, in MD studies of vesicle fusion mediated by either lung surfactant protein SP-B [47] or by SNARE complexes [48], the proteins are observed to trigger spontaneous fusion events by anchoring two vesicles and facilitating the formation of a lipid bridge between the proximal leaflets. Also of interest is a coarse-grained simulation study of another small antimicrobial cyclic peptide, RRKWLWLW [49]. At high enough concentrations, coating of the membrane caused extrusion of lipids from the exposed bilayer leaflet, leading ultimately to a release of phospholipid micellar aggregates (in this study no apposing membrane was present). Thus, destabilization of lipids by membrane active peptides appears to be a generic feature.

Interestingly, concurrent with the formation of the membrane bridge, our MD simulations show that a pore is induced in the lipid membrane, pointing to a dual role of the BPC194. Pore formation is generally viewed as the main mode of action of antimicrobial peptides leading to cell content leakage or even complete lysis of the cell membrane. However, we speculate that under conditions where membranes are in close proximity, both stalks and pores can be formed as another way to relieve the lipid stress caused due to asymmetric peptide binding. A system with both fusion stalks and adjacent pores (distinct from the fusion pores) has been termed a stalk/pore complex and has recently been shown to represent a key intermediate in a possible fusion pathway [50]. Although apposing membranes are pre-established in our simulation studies, they also occur in our in vitro studies in which liposomes are found to aggregate, possibly as a result of the bridging peptides. The stalk/pore pathway is distinct from the traditional pathway of fusion, which proceeds via the formation of a stalk that expands in a radial way forming a hemifusion diaphragm [50]–[54]. Fusion is completed when the hemifusion diaphragm ruptures (via a fusion pore). In the stalk/pore pathway, one or more pores appear in the vicinity of the stalk, allowing propagation of the stalk along the edge of the pore. Upon closure, the HD state is reached, or, in the case when two pores have formed, full fusion is accomplished. Such a pathway has been predicted to be energetically favorable based on mean field calculations [55]. In fact, in MD simulations, the stalk/pore complex is stabilized by fusion peptides, and the peptides and lipids form an inverted cubic phase consisting of a network of stalk/pore complexes [56], [57]. The pores seen in our simulations in the proximity of the membrane bridge is reminiscent of such a pathway and may be considered an intermediate prior to a stalk/pore complex. Thus, we believe that BPC194 can lower the energy barrier towards fusion by stabilizing the stalk/pore complex. The experimental work also suggests that AMPs can stabilize non-lamellar phases, and particularly inverted cubic phases [58]. The ability of AMPs to induce saddle-splay curvature has furthermore been linked to the lipid composition of the membrane, and has been implicated to be a generic mechanism for formation of pores, blebs, buds, and tubes [59]. These results point to a stabilization of saddle-splay (Gaussian) curvature by membrane adsorbed peptides. In this respect, BPC194, a synthetic cyclic antimicrobial peptide may act similar to fusion peptides.

### Conclusions

In conclusion, by using *in vivo, in vitro* and *in silico* methods, we have established that fusion and poration are correlated in the case of BPC194, a synthetic antimicrobial peptide. This dual action is most likely functionally relevant and may contribute to the high potency towards bacterial killing. As probed by DCFBA and optical imaging, poration and fusion occur simultaneously at the same concentration regimes. In fact, in the DCFBA profiles, an increase of leakage coincides qualitatively with an increase in membrane fusion. Cryo-TEM corroborates the fusogenic action of the peptide. The MD simulations furthermore show that pores and stalk-like membrane bridges are formed simultaneously. We believe that the interaction of the cyclic antimicrobial peptide BPC194 with bilayers promotes saddle-splay curvature that is required for both stalks and pores. In case of isolated membranes, pores are formed, but in case membranes are in close proximity the peptides are able to bridge them. This, in turn, leads to the formation of a stalk/pore complex, which is an on-pathway intermediate for membrane fusion. Together, these results explain the dual action of cyclic peptides causing both fusion and leakage. The results are consistent with the whole cell studies, which correlate membrane reorganization with bacteriostasis. Based on our current work and recent studies in other groups [47], [48], [56], [57], [60], we believe that the stalk/pore pathway could be a common mode of action of membrane active peptides.

## Supporting Information

Movie S1 **Cell growth control.** Imaging of cells growing without peptide. Scale bar is 10 µm.
(AVI)

Movie S2 **Cell growth inhibition by BPC194.** Cell growth in the presence of 12.5 µM BPC194. Imaging by light microscopy. Scale bar is 10 µm.
(AVI)

Movie S3 **Cell growth in the presence of BPC193.** Imaging of cells growing in the presence of 12.5 µM of BPC193 by light microscopy. Scale bar is 10 µm.
(AVI)

Movie S4 **Time course of stalk and pore formation by BPC194.** At the beginning all the peptides are between the two bilayers. As the simulations start, some of the peptides bind to one of the bilayers while the remaining peptides bridge both proximal leaflets. As a result, the lipids are perturbed locally and protrude from the bilayer and a stalk is formed. The peptides are depicted in pink, the phosphorous atoms in yellow and green respectively and the lipid chains in grey.
(AVI)

Movie S5 **Lipid splaying caused by cyclic AMPs upon interaction with membranes.** As the lipids come in close contact with the cyclic peptides large perturbations occur. Lipid splay occurs and is helped by the interaction between the lysine residues and the charged lipid head-group as well as the phenylalanine and leucine residues with the hydrophobic tails of the lipid. The peptides’ backbone is shown in pink, lysine residues in orange, the phenylalanine and leucine residues in white, the phosphorous atom in green and lipid chain in grey. For clarity, average density of the lipid molecules in the two leaflets in the stalk phase is shown in yellow and green, respectively.
(AVI)

Supporting Information S1 (DOC)
