# Supplementary material for: Dual Action of BPC194: A Membrane Active Peptide Killing Bacterial Cells
Source: PLoS One. 2013 Apr 19;8(4):e61541. doi: 10.1371/journal.pone.0061541 (PMC3631201; doi:10.1371/journal.pone.0061541)
Supplement: Supporting Information S1 — (DOC) [file pone.0061541.s006.doc]

**Supporting Information**

Dual action of BPC194: A membrane active peptide killing bacterial cells

Gemma Moiset^1,§^, Anna D. Cirac^1,2,§^, Marc C. A. Stuart^1^, Siewert J. Marrink^1^, Durba Sengupta^1,3,*^, and Bert Poolman^1,*^

^1^Departments of Biochemistry and Biophysical Chemistry, Groningen Biomolecular Sciences and Biotechnology Institute (GBB) and Zernike Institute for Advanced Materials, University of Groningen, Nijenborgh 4, 9747 AG Groningen, the Netherlands

^2^Institute of Computational Chemistry, University of Girona, Campus Montivili, 17071 Girona, Spain

^3^Physical Chemistry Division, CSIR-National Chemical Laboratory, Pune-411008, India

*To whom correspondence may be addressed. E-mail: b.poolman@rug.nl or d.sengupta@ncl.res.in

^§^These authors contributed equally to this work

**SUPPORTING METHODS**

Peptide stability followed by High-Performance Liquid Chromatography (HPLC) – Escherichia coli (E. coli) K-12 strain MC1061 was grown from single colonies in LB at 37ºC under vigorous aeration until the culture reached an OD_600_ of 0.1. A 1 mL volume of cells was pellet by centrifugation (4 min; 10,000 x g; room temperature) and resuspended in 1 mL 10 mM potassium phosphate, 150 mM NaCl, pH 7 (equiosmolar with LB medium) supplemented with 100 µM cyclic peptide, BPC194, or linear homologue, BPC193. Aliquots were taken after 5, 10, 30 and 60 min of incubation at r.t., and samples were centrifuged. Cell pellets and supernatants were kept at 4ºC until they were analyzed by HPLC. Reversed-phase HPLC with UV detection was performed on a Shimadzu system consisting of two LC-20AD pumps, a SPD-M20A detector, a SIL-20AC HT auto-sampler and a CBM-20A system controller (Shimadzu, Kyoto, Japan). The peptides in the supernatant solutions were separated on a XTerra MS C18 column (3 × 150 mm) (Waters). The column temperature was set to 35ºC. Analyses were performed using a linear gradient of 5–95% eluent B (acetonitrile with 0.1% TFA; eluent A was water with 0.1% TFA) at a ﬂow rate of 0.5 mL/min over 40 minutes. UV detection was carried out at 220 nm (PDA spectra 190–700 nm). The reversed-phase HPLC retention time of each peptide was determined when the peak was at its maximum height, and the area of the peak was used to determine the (relative) concentration of the peptides (using the appropriate standards and time zero samples). Since a fraction of the peptides is bound to cells, we treated cell pellets with 6M urea (final concentration) plus 1.5% (v/v) Triton X-100. After 30 min of extraction, the sample was centrifuged and the supernatant was analyzed by HPLC. In control experiments, standard samples of peptide were incubated with urea plus Triton X-100 in the same manner.

**SUPPORTING FIGURE**


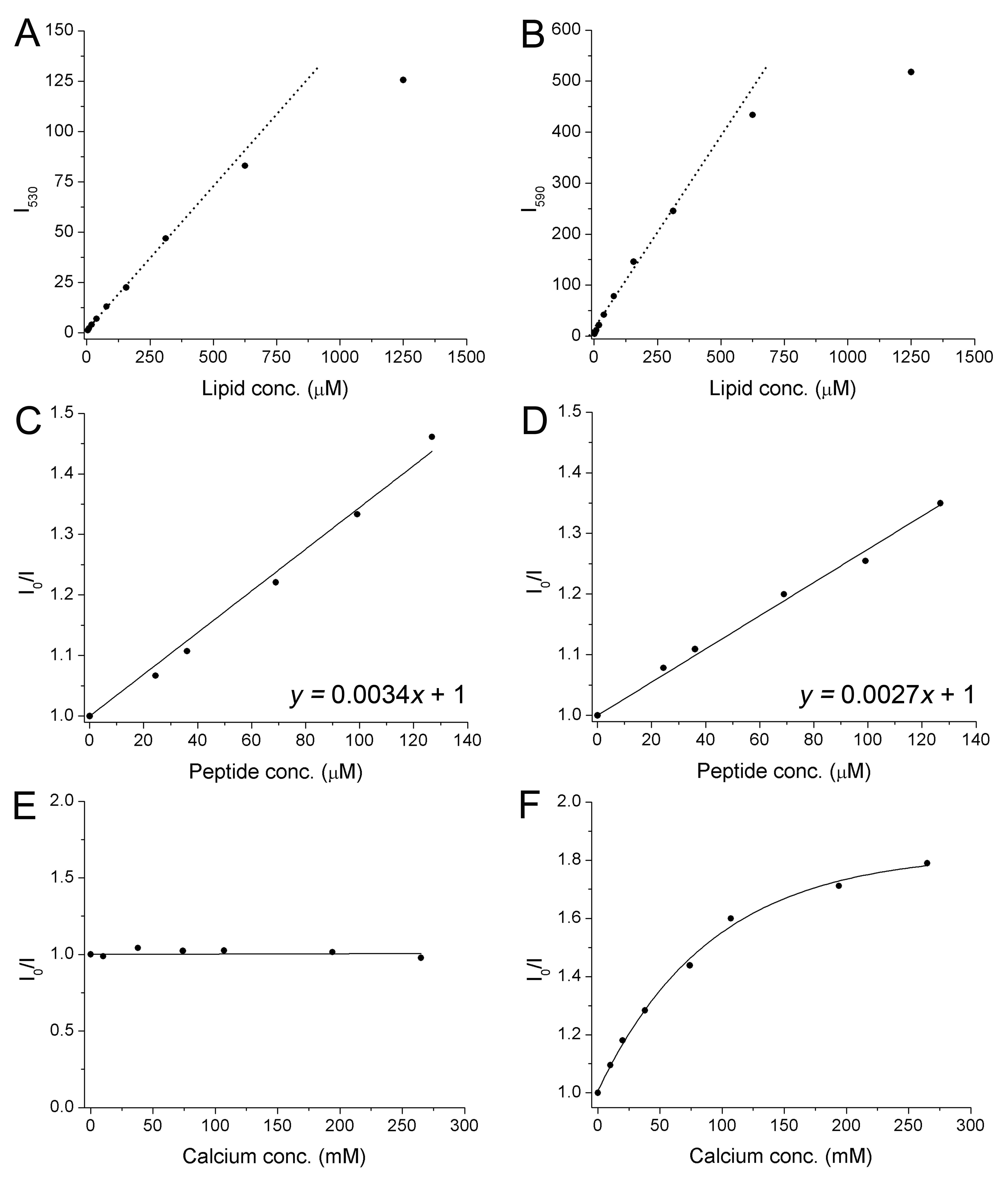


**Fig. S1.** **Controls for inner filter effect and quenching of fluorophores by peptide and calcium.** A-B. NBD and Rhodamine fluorescence as a function of lipid concentration. The dotted lines show the linear regime where inner filter effects are not present. C-D. NBD and Rhodamine fluorescence as a function of peptide concentration. The dependence of I_0_/I on peptide concentration (see equation) was used to correct for the quenching. E-F. NBD and Rhodamine fluorescence as function of calcium concentration. Calcium does not quench NBD emission intensity but does quench the Rhodamine fluorescence.

**
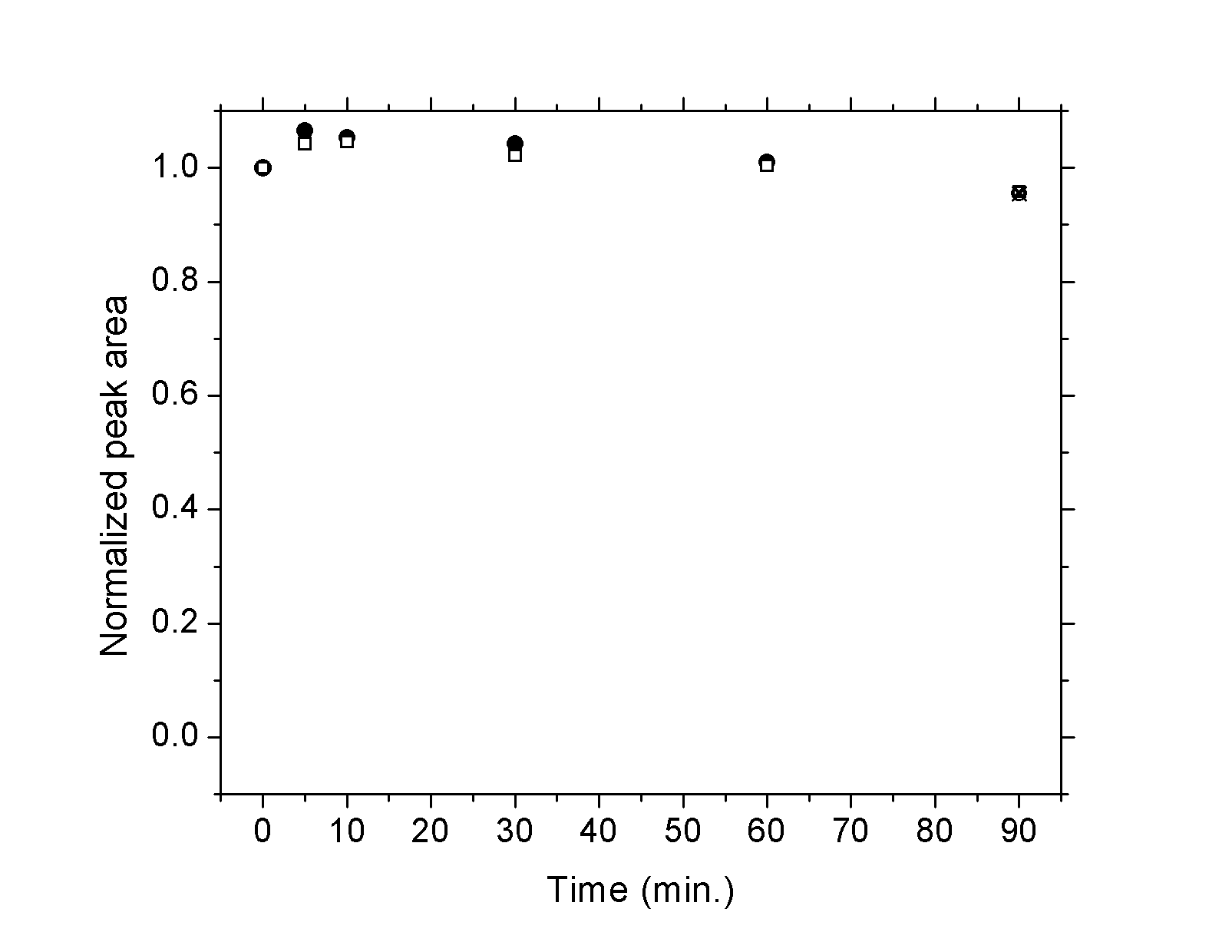
**

**Fig. S2.** **Stability of peptides.** Normalized peak area of BPC194 (full circles) and BPC193 (empty squares) after incubation with *E. coli* cells for different periods of time. The data points at 90 min. (depicted with a crossed-circle or –square for BPC194 or BPC193, respectively) represent the aliquots incubated for 60 min with cells and subsequently another 30 more min in the presence of 6M urea plus 1.5% (v/v) Triton X-100. Peptides not exposed to cells but incubated with urea plus Triton X-100 did not show any decrease in the peak area (data not shown). The retention times were 20.0 min for BPC193 and 23.4 min for BPC194. As is evident from the data, we did not observe significant breakdown of either BPC193 or BPC194 upon incubation with *E. coli* cells up to a period of at least 1h.

**
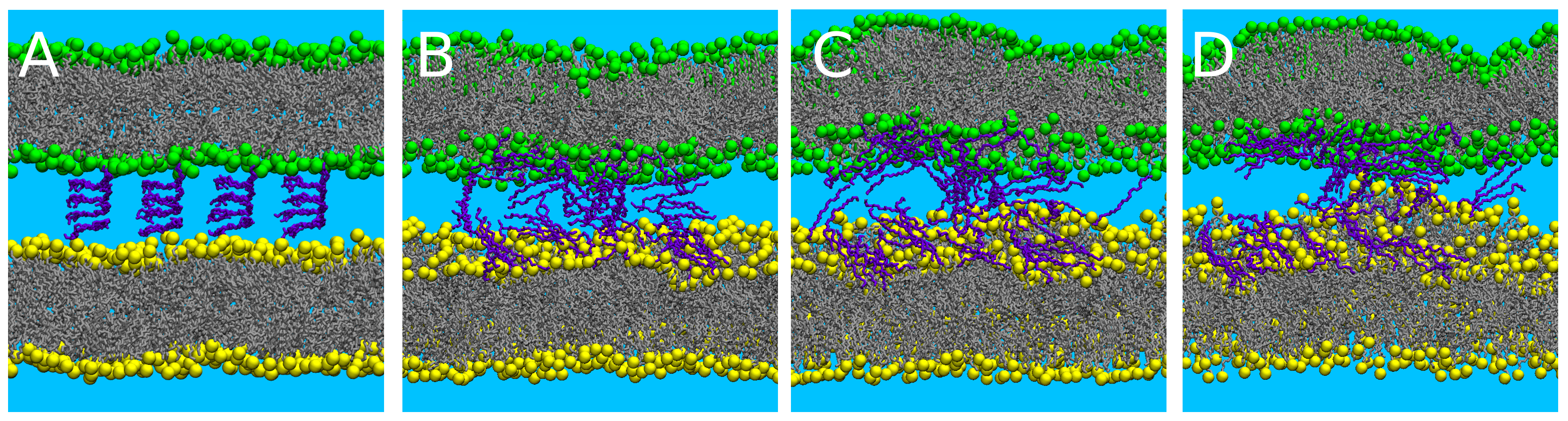
**

**Fig. S3. MD simulation of the inactive linear peptide in contact with a membrane.** A: Initial simulation setup of the linear analogue (BPC193), which is the same as for the cyclic peptide BP194 (0 ns). B-D: From 8 to 70 ns of the simulation, the linear peptides are not able to perturb the membranes and induce fusion. Poration or pre-stalk events are not observed.

**SUPPORTING MOVIES: STILL IMAGES**


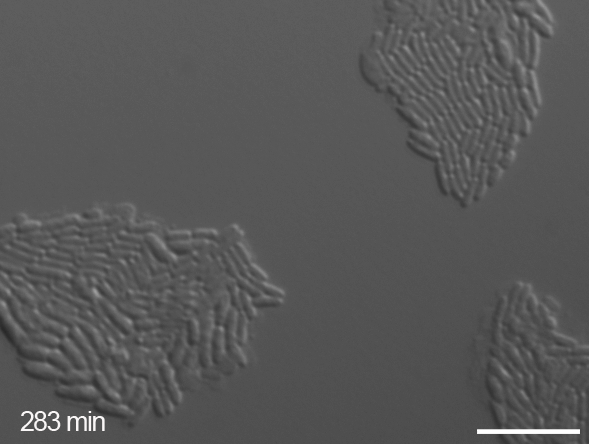


**Movie 1.** *Cell growth control*. Imaging of cells growing without peptide. Scale bar is 10 µm.


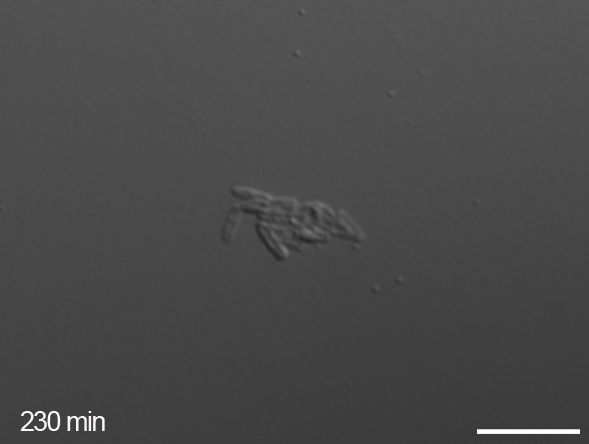


**Movie 2.** *Cell growth inhibition by BPC194*. Cell growth in the presence of 12.5 µM BPC194. Imaging by light microscopy. Scale bar is 10 µm.


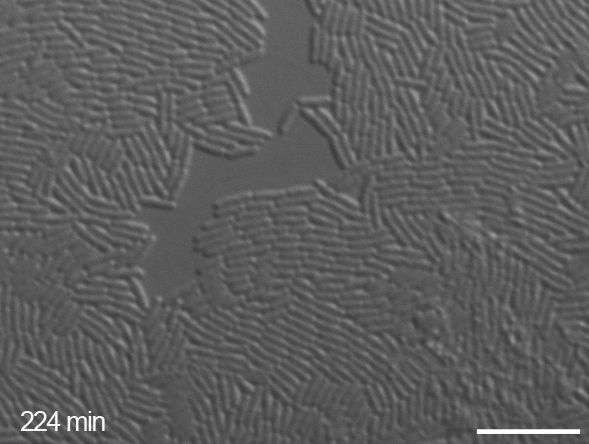


**Movie 3.** *Cell growth in the presence of BPC193.* Imaging of cells growing in the presence of 12.5 µM of BPC193 by light microscopy. Scale bar is 10 µm.


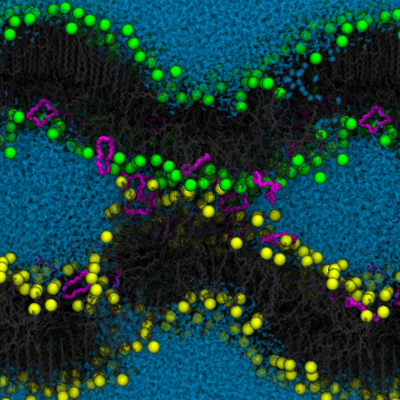


**Movie 4.** *Time course of stalk and pore formation by BPC194.* At the beginning all the peptides are between the two bilayers. As the simulations start, some of the peptides bind to one of the bilayers while the remaining peptides bridge both proximal leaflets. As a result, the lipids are perturbed locally and protrude from the bilayer and a stalk is formed. The peptides are depicted in pink, the phosphorous atoms in yellow and green respectively and the lipid chains in grey.


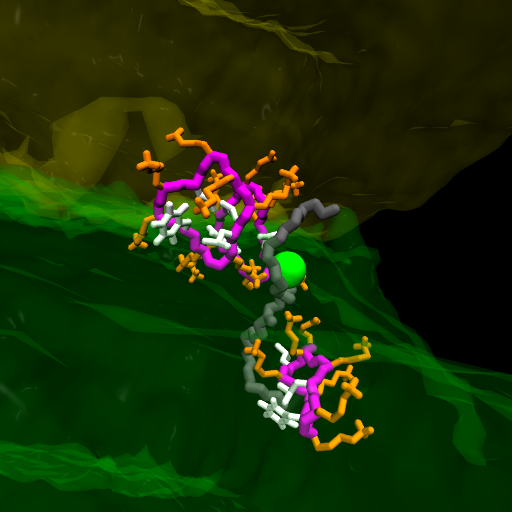


**Movie 5.** *Lipid splaying caused by cyclic AMPs upon interaction with membranes.* As the lipids come in close contact with the cyclic peptides large perturbations occur. Lipid splay occurs and is helped by the interaction between the lysine residues and the charged lipid head-group as well as the phenylalanine and leucine residues with the hydrophobic tails of the lipid. The peptides' backbone is shown in pink, lysine residues in orange, the phenylalanine and leucine residues in white, the phosphorous atom in green and lipid chain in grey. For clarity, average density of the lipid molecules in the two leaflets in the stalk phase is shown in yellow and green, respectively.
